# Use of *Salmonella enterica* Serovar Typhi Hemolysin E and Lipopolysaccharide IgA to Identify Enteric Fever Cases, South Asia

**DOI:** 10.3201/eid3208.250753

**Published:** 2026-08

**Authors:** Jessica C. Seidman, Kristen Aiemjoy, Mehreen Adnan, Irum Fatima Dehraj, Junaid Iqbal, Khalid Iqbal, Seema Irfan, Nahidul Islam, Md. Shakiul Kabir, Nishan Katuwal, Noshi Maria, Muhammad Ashraf Memon, Sira Jam Munira, Shiva Ram Naga, Sailesh Pradhan, Anik Sarkar, Rajeev Shrestha, Sony Shrestha, Syed Muktadir Al Sium, Krista Vaidya, Douglas Ezra Morrison, Alice S. Carter, Senjuti Saha, Dipesh Tamrakar, Mohammad Tahir Yousafzai, Denise O. Garrett, Stephen P. Luby, Farah Naz Qamar, Samir Saha, Jason R. Andrews, Richelle C. Charles

**Affiliations:** Sabin Vaccine Institute, Washington, DC, USA (J.C. Seidman, A.S. Carter, D.O. Garrett); Mahidol University Faculty of Tropical Medicine, Bangkok, Thailand (K. Aiemjoy); University of California, Davis, California, USA (K. Aiemjoy, K. Vaidya, D.E. Morrison); Aga Khan University, Karachi, Pakistan (M. Adnan, I.F. Dehraj, J. Iqbal, S. Irfan, N. Maria, M.T. Yousafzai, F.N. Qamar); Kharadar General Hospital, Karachi (K. Iqbal, M.A. Memon); Child Health Research Foundation, Dhaka, Bangladesh (N. Islam, M.S. Kabir, S.J. Munira, A. Sarkar, S.M. Al Sium, Senjuti Saha, Samir Saha); Dhulikhel Hospital Kathmandu University Hospital, Kavrepalanchok, Nepal (N. Katuwal, S.R. Naga, R. Shrestha, S. Shrestha, D. Tamrakar); Kathmandu Medical College and Teaching Hospital, Kathmandu, Nepal (S. Pradhan); Bangladesh Council of Scientific and Industrial Research, Dhaka (S.M. Al Sium); Stanford University, Stanford, California, USA (S.P. Luby, J.R. Andrews); Massachusetts General Hospital, Boston, Massachusetts, USA (R.C. Charles); Harvard Medical School, Boston (R.C. Charles); Harvard T.H. Chan School of Public Health, Boston (R.C. Charles)

**Keywords:** enteric infections, bacteria, Salmonella Typhi, Salmonella Paratyphi, Salmonella enterica, South Asia, Bangladesh, Nepal, Pakistan

## Abstract

Existing methods to identify patients infected with *Salmonella enterica* serovar Typhi or Paratyphi are not adequately accurate, affordable, or efficient. We evaluated the performance of antibodies to *Salmonella* Typhi hemolysin E (HlyE) and lipopolysaccharide (LPS) in Bangladesh, Nepal, and Pakistan for enteric fever case identification. We measured plasma concentrations of HlyE and LPS IgA in blood culture–confirmed enteric fever case-patients and in febrile controls with laboratory-confirmed alternative etiologies. Combining LPS and HlyE IgA discriminated enteric fever cases from other febrile illnesses with an area under the receiver operating characteristic curve (AUC) of 0.93 (specificity 86% at a fixed 90% sensitivity). LPS IgA alone performed nearly as well (AUC 0.92). In children <5 years of age, the combined biomarkers outperformed either biomarker alone (AUC 0.96 vs. 0.94 for HlyE, 0.93 for LPS). Our findings support use of HlyE and LPS IgA–based assays for enteric fever diagnosis in endemic settings.

Enteric fever is a systemic infection caused by gram-negative bacteria *Salmonella enterica* serovars Typhi and Paratyphi. Enteric fever is transmitted through contaminated food and water and is common where sanitation is inadequate (*1*). Accurate diagnosis is challenged by nonspecific clinical manifestation and poor-performing diagnostics (*2*–*4*). Definitive identification requires *Salmonella* Typhi or Paratyphi isolation from blood or bone marrow, which is performed in a microbiology laboratory and produces results after several days. Blood culture is highly specific but poorly sensitive, expensive, slow, and widely inaccessible where the disease is most common (*5*,*6*); the Widal serum-agglutination test is frequently used despite poor accuracy in endemic settings (*7*). In a previous evaluation, commercial typhoid rapid diagnostics failed to perform with both sensitivity and specificity >90% (*8*).

Two antigens, pore-forming cytotoxin hemolysin E (HlyE) and *Salmonella* Typhi lipopolysaccharide (LPS), have shown promise for typhoid serodiagnostic assays; previous studies were limited by small sample size and narrow geographic scope (*9*–*11*). In a pediatric cohort in Nigeria, LPS and HlyE-specific IgA provided good discrimination between Typhi and other bacteremias; the receiver operator characteristic (ROC) area under the curve (AUC) for LPS-specific IgA was 0.90 and for HlyE-specific IgA was 0.74 (*12*). In previous work studying *Salmonella* Typhi cases and other bacteremias in Nepal, we found the combination of HlyE and LPS IgA was 90% sensitive and 92% specific (AUC 0.95) (*9*). Those studies did not include *Salmonella* Paratyphi A cases; controls had primarily other bacteremias or no etiology identified. The same biomarkers demonstrated high accuracy in a dual-antigen lateral flow format in Pakistan (AUC 0.93) (*13*) and Bangladesh (AUC 0.97) (*14*). 

The multisite SeroEpidemiology and Environmental Surveillance (SEES) Study measured longitudinal HlyE and LPS antibody responses in blood culture–positive enteric fever cases and estimated enteric fever seroincidence in disease-endemic communities in Bangladesh, Nepal, and Pakistan (*15*). In this substudy, we evaluated the performance of HlyE and LPS IgA to distinguish clinically manifesting enteric fever case-patients from acute febrile patients with confirmed alternative etiologies. We also assessed how the dynamics of convalescent antibody responses could influence assay performance in high-incidence settings. 

## Materials and Methods

### Study Participants and Procedures

We recruited participants from a hospital-based enteric fever surveillance study in Dhaka, Bangladesh (Bangladesh Shishu Hospital & Institute); Kathmandu and Kavrepalanchowk, Nepal (Kathmandu Medical College and Teaching Hospital, Dhulikhel Hospital, and Kathmandu University Hospital); and Karachi, Pakistan (Aga Khan University Hospital and Kharadar General Hospital) (*16*). Prospectively and retrospectively enrolled cases were eligible if they had positive blood culture for *Salmonella* Typhi or Paratyphi A, were enrolled in the SEES study, and had a baseline plasma sample (*15*). We enrolled cases during October 2017–June 2021 in Bangladesh, March 2017–April 2021 in Nepal, and May 2019–June 2022 in Pakistan. Participants were eligible as alternative etiology controls if they experienced fever of 3 days as outpatient or any duration as inpatient and had laboratory confirmation of another etiology (Appendix Table 1). We enrolled controls during June 2019–July 2021 in Bangladesh, October 2016–July 2021 in Nepal, and May 2019–June 2022 in Pakistan. Patients with multiple pathogens detected could be included as controls unless blood culture was positive for *Salmonella* Typhi or Paratyphi within 30 days of enrollment; blood cultures were not performed on all controls.

We obtained written informed consent from all adult participants (>18 years of age) and from a parent or guardian for children <18 years; participants 15–17 years of age also provided written assent. We captured patient demographics and symptom history in a structured questionnaire.

The ethics review committees of collaborating institutions approved this study: Bangladesh Institute of Child Health (no. BICH-ERC-01/02/2019), Nepal Health Research Council for Dhulikhel Hospital (no. 391/2018), Aga Khan University (no. 2019-0410-4188), Pakistan National Bioethics Committee (no. 4-87/NBC-341-Amend-revised/19/81), Stanford University (no. 39557), and MassGeneral Brigham (no. 2019P000152).

### HlyE and LPS IgA ELISAs

Plasma separated from whole blood by centrifugation was stored at −70°C until testing. To measure the concentration of IgA antibodies to HlyE and LPS IgA, we used kinetic ELISA as previously described (*15*); we reported results as ELISA units (EUs) (Appendix Figure 1).

### Statistical Analyses

We limited inclusion to participants <50 years of age reporting <14 days of fever when they sought care for whom we had complete ELISA results. We evaluated performance of HlyE IgA and LPS IgA individually and in combination with ROC analysis; we combined the biomarkers by logistic regression (*9*). We assessed biomarker performance with AUC overall and in stratified analyses by site, age, and age plus site categories and by *Salmonella* serovar. We calculated sensitivity, specificity, and associated 95% CIs by bootstrapping; we compared AUCs using DeLong’s method (*17*,*18*) (Appendix). Sensitivity analyses removed the inclusion age restriction and stratified by fever duration at the time of presentation to care.

To estimate the duration of postinfection positive test results using these biomarkers, we assessed when convalescent antibody concentrations declined below positivity thresholds defined post hoc. We identified single biomarker cutpoints by maximizing Youden’s J statistic; for joint biomarker cutpoints we identified the paired thresholds that maximized balanced accuracy (Appendix). We characterized postinfection longitudinal antibody dynamics using hierarchical 2-phase within-host models (*15*,*19*). The model distinguished between an initial active infection period, with an exponential antibody rise, and nonexponential decay period after pathogen elimination. We measured time since self-reported fever onset. Using a Bayesian framework, we jointly estimated model parameters and hyperparameters with Markov chain Monte Carlo sampling; we then evaluated when median longitudinal responses fell below individual and joint biomarker cutpoints.

We performed all analyses using R version 4.3.3 (The R Project for Statistical Computing, https://www.r-project.org); we calculated ROC curves, cutpoints and associated performance metrics, and CIs using the cutpointR package version 1.1.2 (https://cran.r-project.org/web/packages/cutpointr). We estimated fixed sensitivity and specificity and associated CIs using the pROC package version 1.18.5 (https://cran.r-project.org/web/packages/pROC). We implemented Markov chain Monte Carlo sampling using JAGS version 4.3.2 (https://cran.r-project.org/web/packages/rjags).

## Results

### Participant Characteristics

**Figure 1:**
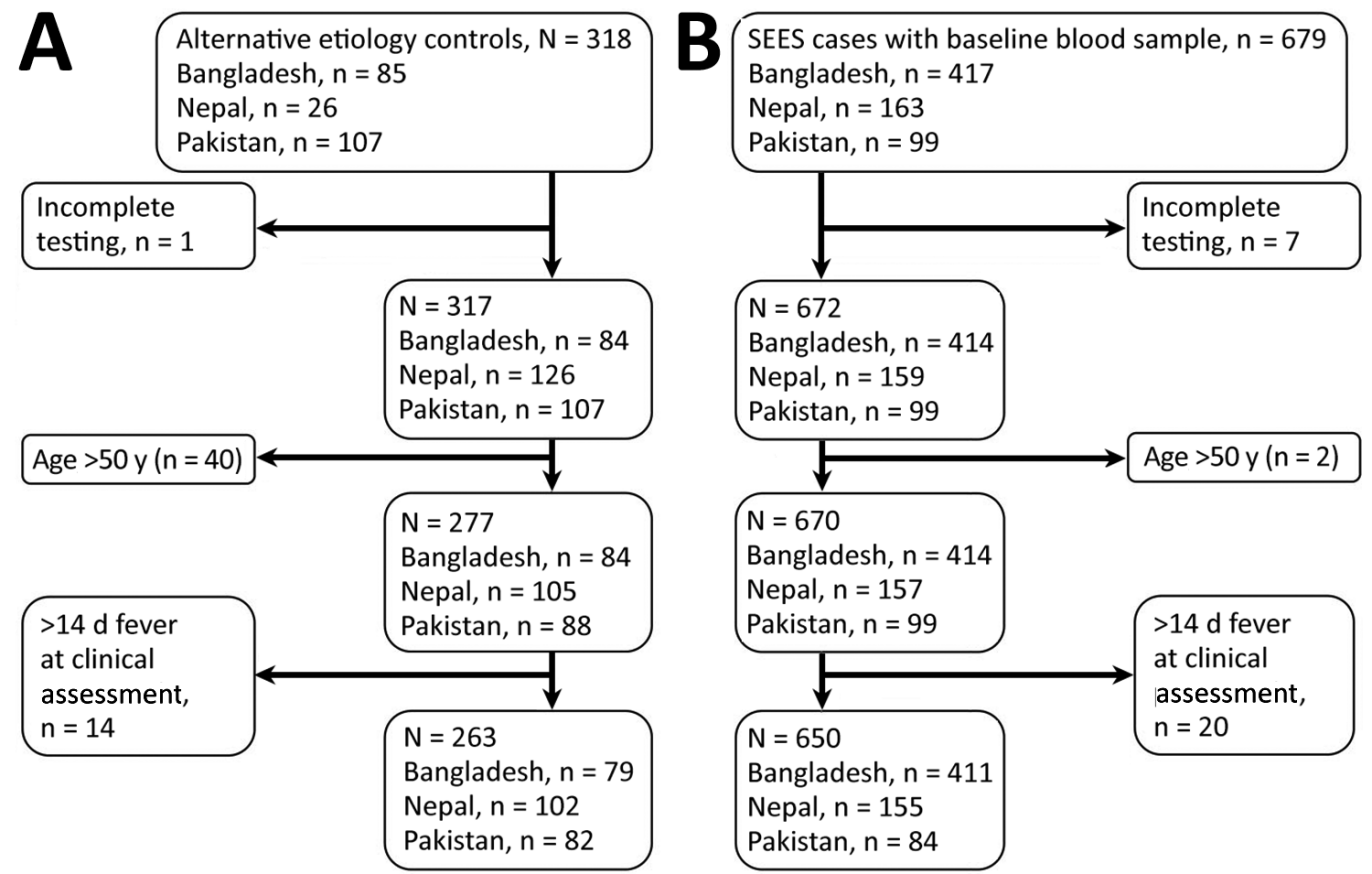
Flowchart of inclusion and exclusion of participants in study of the use of *Salmonella* Typhi hemolysin E and lipopolysaccharide IgA to identify enteric fever cases, South Asia. Controls (A) and cases (B) were recruited from 6 hospitals in Dhaka, Bangladesh; Kathmandu Valley, Nepal; and Karachi, Pakistan. SEES, SeroEpidemiology and Environmental Surveillance study.

From the SEES study, 679 blood culture–positive *Salmonella* Typhi and Paratyphi A cases had a baseline blood sample; 650 of those were eligible for inclusion in our substudy (n = 568 for Typhi and n = 82 for Paratyphi A) (Figure 1). In Bangladesh (N = 411), the median patient age was 5.4 (IQR 3.3–8.0) years and 48% were female and 53% male; in Nepal (N = 155) the median age was 19.4 (IQR 14.9–25.7) years and 39% were female and 61% male; and in Pakistan (N = 84) the median age was 9.0 (IQR 4.0–17.0) years and 43% were female and 57% male (Table).

**Table:** Characteristics of laboratory-confirmed enteric fever cases and alternative etiology controls in study of the use of *Salmonella* Typhi hemolysin E and lipopolysaccharide IgA to identify enteric fever cases, South Asia*

Characteristic	Bangladesh			Nepal			Pakistan			All		p value
	Case, n = 411	Control, n = 79		Case, n = 155	Control, n = 102		Case, n = 84	Control, n = 82		Case, n = 650	Control, n = 263	
Age												
Median	5.4	3.0		19.4	28.0		9.0	29.5		7.0	22.0	<0.001
IQR, Q1–Q3	3.3–8.0	1.0–7.0		14.9–25.7	19.5–38.0		4.0–17.0	17.0–39.8		4.0–13.0	4.5–34.0	
Age category, y, no. (%)												
<5	180 (43.8)	50 (63.3)		4 (2.6)	7 (6.9)		24 (28.6)	9 (11.0)		208 (32.0)	66 (25.1)	<0.001
5–15	230 (56.0)	28 (35.4)		39 (25.2)	13 (12.7)		36 (42.9)	10 (12.2)		305 (46.9)	51 (19.4)	
≥16	1 (0.2)	1 (1.3)		112 (72.3)	82 (80.4)		24 (28.6)	63 (76.8)		137 (21.1)	146 (55.5)	
Sex, no. (%)												
M	214 (52.1)	45 (57.0)		95 (61.3)	54 (52.9)		48 (57.1)	69 (84.1)		357 (54.9)	168 (63.9)	0.02
F	197 (47.9)	34 (43.0)		60 (38.7)	48 (47.1)		36 (42.9)	13 (15.9)		293 (45.1)	95 (36.1)	
Days of fever at presentation												
Median	4.0	3.0		4.0	3.0		6.0	5.0		4.0	4.0	0.15
IQR, Q1–Q3	3.0–6.0	2.0–4.0		3.0–5.0	3.0–6.0		4.8–8.0	4.0–7.0		3.0–6.0	3.0–6.0	
HlyE IgA concentration												
Median	27.6	2.1		19.2	4.7		36.5	3.0		26.4	3.4	<0.001
IQR, Q1–Q3	10.4–57.3	0.8–5.5		5.9–50.0	2.6–8.1		15.9–102.3	1.5–5.0		9.5–59.1	1.6–6.3	
LPS IgA concentration												
Median	82.3	2.3		106.1	4.5		162.6	6.6		98.0	4.5	<0.001
IQR, Q1–Q3	31.9–158.9	0.5–5.6		32.6–223.8	3.1–9.8		73.5–321.0	3.6–10.9		35.3–190.2	2.2–9.4	

*p values estimated for the comparisons between all cases and controls. IQR, interquartile range; Q, quarter.

We recruited 318 febrile controls with laboratory-confirmed non–enteric fever etiology. After applying the age and fever inclusion criteria, we analyzed 263 controls (79 from Bangladesh, 102 from Nepal, and 82 from Pakistan). Etiologies identified were dengue (n = 128), COVID-19 (n = 45), malaria (n = 12), scrub typhus (*Orientia tsutsugamushi,* n = 12), gram-negative bacteremia (*Escherichia coli, Klebsiella pneumoniae, Acinetobacter* spp., nontyphoidal *Salmonella* spp*.*, *Proteus mirabilis*, *Enterobacter cloacae*; n = 40) and gram-positive bacteremia (*Staphylococcus aureus, Streptococcus* spp.; n = 26). Of the controls, 43 (16%) lacked blood culture: 29 participants with dengue, 12 with COVID, and 2 with malaria. Across sites, controls were significantly older than case-patients (median age 22 years vs. 7 years; p<0.001).

**Figure 2:**
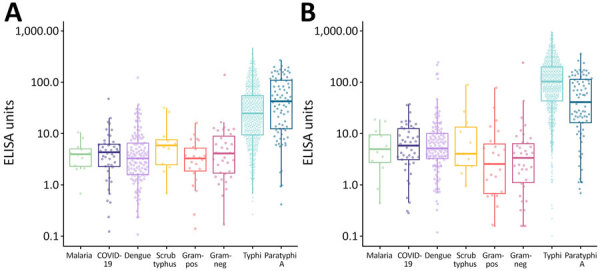
Distribution of HlyE and LPS IgA among febrile cases by pathogen type in study of the use of *Salmonella* Typhi hemolysin E and lipopolysaccharide IgA to identify enteric fever cases, South Asia. Boxplots show the distribution of plasma IgA responses against HlyE and LPS among *Salmonella* Typhi (n = 568) and Paratyphi A (n = 82) cases (A) and controls (B). Controls were febrile patients with dengue (n = 128), COVID-19 (n = 45), malaria (n = 12), scrub typhus (by *Orientia tsutsugamushi*, n = 12), gram-negative bacteria (n = 40), or gram-positive bacteria (n = 26). Dots indicate participants. Upper and lower bounds of each box represent interquartile range (quarter 1–3); horizontal line indicates median; whiskers represent 95% CIs. Neg, negative; pos, positive.

We measured HlyE and LPS IgA plasma responses in febrile enteric fever case-patients and alternative etiology controls at the time they sought care (Table, Figure 2; Appendix Figure 1). Median HlyE and LPS IgA levels for all participants were significantly higher for enteric fever cases than for controls. Median HlyE IgA was 26.4 (IQR 9.5–59.1) EUs in cases versus 3.4 (IQR 1.6–6.3) EUs in controls (p<0.001). Median LPS IgA was 98.0 (IQR 35.3–190.2) EUs in cases versus 4.5 (IQR 2.2–9.4) EUs in controls (p<0.001). 

### Enteric Fever Case Identification Using IgA to HlyE and LPS

**Figure 3:**
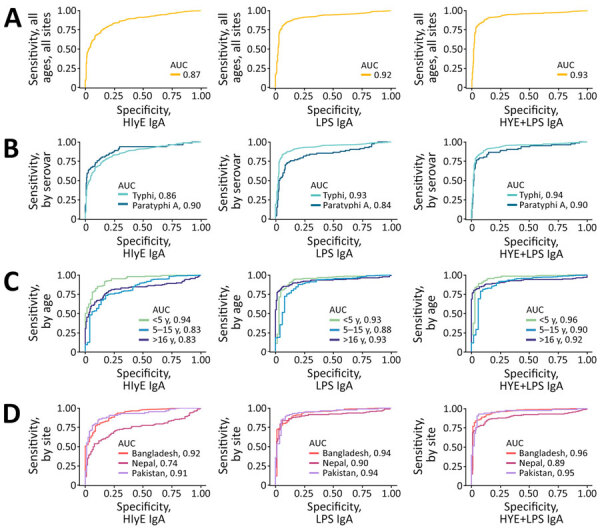
Receiver operating characteristic curves in study of the use of *Salmonella* Typhi hemolysin E and lipopolysaccharide IgA to identify enteric fever cases, South Asia. We analyzed plasma IgA to *Salmonella* Typhi HlyE and LPS alone and in combination to identify enteric fever cases. Receiver operating characteristic and AUC are shown for the complete study population (A) and by *Salmonella* serovar (Typhi or Paratyphi; B), age category (C), and study site (D). AUC, area under the receiver operating characteristic curve.

We assessed classification accuracy of HlyE and LPS IgA overall and in stratified analyses by serovar, age, study site, and age plus site (Appendix Tables 2, 4). In ROC analyses of the overall study population, the combined biomarker model yielded an AUC of 0.93 (95% CI 0.91–0.95); at a fixed sensitivity of 90%, specificity was 86% (95% CI 0.76–0.91) (Figure 3; Appendix Table 3). The combined biomarker model performed better than either biomarker individually, but LPS IgA was nearly as accurate as a solo biomarker: LPS AUC 0.92 (95% CI 0.90–0.94); HlyE + LPS versus LPS AUC difference 0.02 (95% CI 0.01–0.02; p<0.001); HlyE AUC 0.87 (95% CI 0.84–0.89); HlyE + LPS versus HlyE AUC difference 0.06 (95% CI 0.04–0.08; p<0.001).

### Biomarker Performance by Serovar

The combined biomarker model was also slightly better at identifying *Salmonella* Typhi (AUC 0.94 [95% CI 0.92–0.95]) versus Paratyphi A (AUC 0.90 [95% CI 0.86–0.95]; AUC difference 0.03 [95% CI −0.02 to 0.08; p = 0.18]) infections, although that difference was not statistically significant (Appendix Table 4). However, as individual biomarkers, HlyE IgA was marginally better at identifying paratyphoid cases than LPS IgA (AUC 0.90 [95% CI 0.85–0.94]) vs. 0.84 [95% CI 0.78–0.90]; AUC difference [0.06 95% CI] 0.01–0.11; p = 0.02). Pakistan had insufficient *Salmonella* Paratyphi A cases (n = 1) for serovar and site-stratified analyses. In Bangladesh and Nepal, the combined biomarker model performed similarly in identifying *Salmonella* Typhi and Paratyphi A cases. As we observed in the pooled analysis, LPS IgA was marginally better at identifying *Salmonella* Typhi versus Paratyphi A cases, but the difference was statistically significant only in Bangladesh (AUC difference 0.07 [95% CI 0.01–0.14]; p = 0.03). In contrast, HlyE IgA was better at identifying Paratyphi A cases in Nepal (AUC difference 0.16 [95% CI 0.04– 0.28]; p = 0.01).

### Biomarker Performance in Age and Site Strata

The biomarker pair worked best in the youngest children, those <5 years of age (AUC 0.96 [95% CI 0.92–0.99]), a slight improvement over either biomarker alone in that age group: HlyE AUC 0.94 (95% CI 0.91–0.97), HlyE plus LPS versus HlyE (AUC difference 0.02 [95% CI −0.01 to 0.04]; p = 0.27); LPS AUC 0.93 (95% CI 0.90–0.97); and HlyE plus LPS versus LPS (AUC difference 0.02 [95% CI 0.01–0.04]; p<0.001). The combined biomarkers also performed differently by site; the Nepal cohort had a lower AUC (0.89 [95% CI 0.85–0.94]) compared with Bangladesh (0.96 [95% CI 0.93–0.98]; AUC difference −0.06 [95% CI −0.11 to −0.01]; p = 0.01) and Pakistan (0.95 [95% CI 0.92–0.99]; AUC difference −0.06 [95% CI −0.12 to −0.01]; p = 0.03). In age plus site–specific strata, the highest AUC was in Bangladesh in children <5 years of age (0.97 [95% CI 0.96–0.99]) and lowest in Nepal in children 5–15 years of age (0.82 [95% CI 0.68–0.96]); however, none of the differences across age plus site–specific strata were statistically significant (p>0.05).

### Sensitivity Analyses

We compared biomarker performance across reported fever of different duration at the time the patient sought care (<3, 4–5, 6–14 days of fever) (Appendix Table 5). The combination of HlyE and LPS IgA best distinguished cases from controls who reported 4–5 days of fever (AUC 0.96 [95% CI 0.94–0.98]). In site-specific strata, we observed no statistically significant differences in accuracy by fever duration using LPS IgA alone or in combination with HlyE. However, when we used HlyE IgA alone, we observed a trend of improved performance with fevers of longer duration in all-site analysis and in Bangladesh and Nepal. 

In our primary analyses, we excluded participants >50 years of age because the control population was substantially older; we removed 2 cases and 21 controls from Nepal and 19 controls from Pakistan. In a sensitivity analysis including those older participants, results were nearly identical to the primary findings (Appendix Table 6).

### Longitudinal Antibody Decay among Convalescent Cases

**Figure 4:**
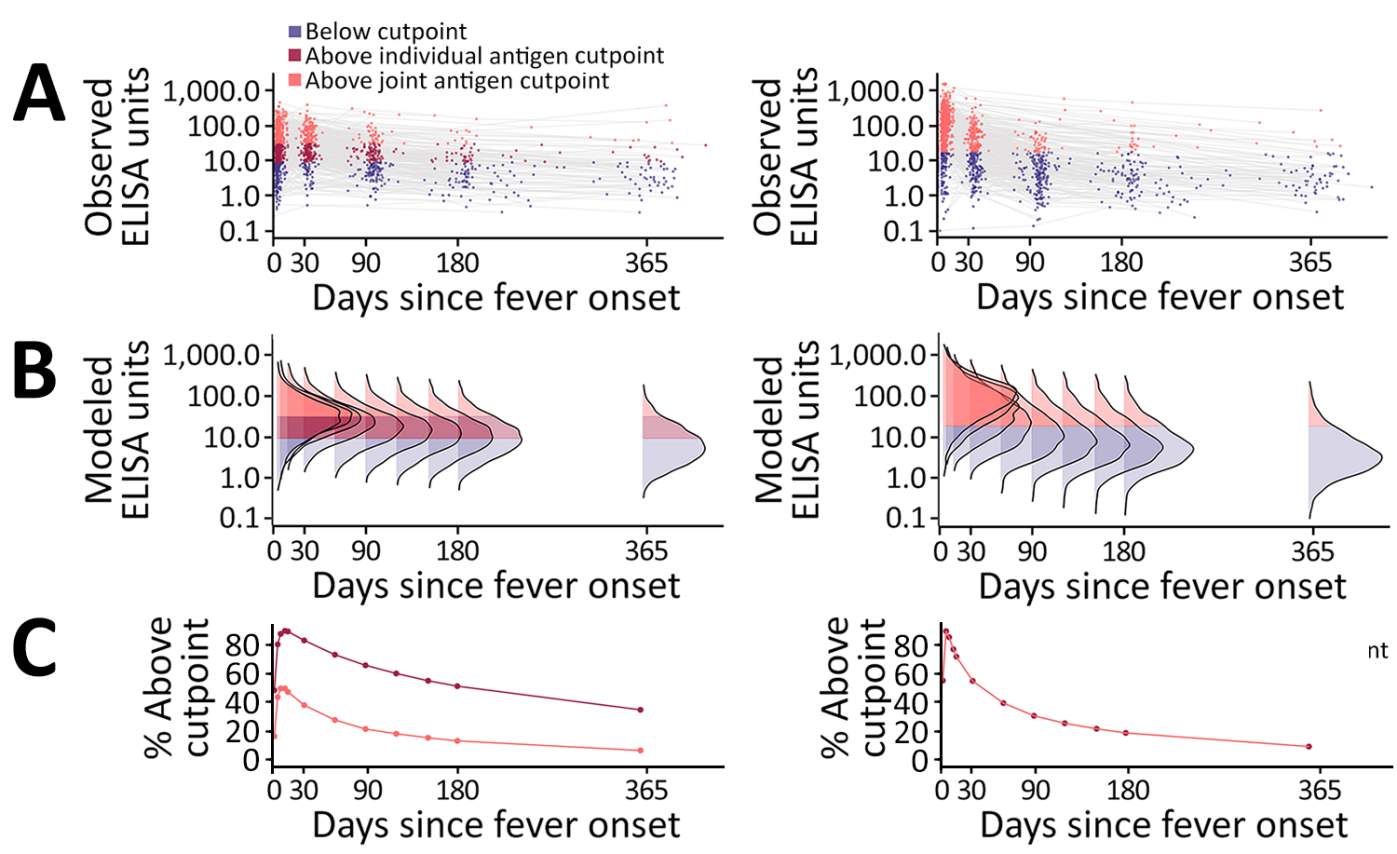
Longitudinal HlyE (left) and LPS (right) IgA responses in enteric fever cases during the year after fever onset in study of the use of *Salmonella* Typhi hemolysin E and lipopolysaccharide IgA to identify enteric fever cases, South Asia. A) Observed antibody responses from blood culture–confirmed enteric fever cases. Colored points represent measured antibody ELISA concentrations and gray lines connect observations from the same participant. B) Distribution of antibody responses from fitted hierarchical 2-phase within-host models during the year after infection. C) Percentage of modeled antibody responses above the individual and joint cutpoints for enteric fever case identification during the year after infection.

Observed and modeled antibody dynamics revealed that LPS IgA peaks earlier and decays more rapidly than HlyE IgA (Figure 4; Appendix Table 7). Those findings are reflected in its larger shape factor (2.35 vs. 2.07) and slightly faster decay rate (0.000355/d vs. 0.000311/d), indicating a more rapid initial decline in antibody concentration. Antibody responses to LPS IgA reached a significantly higher peak concentration (232.6 EUs) at an earlier time point (2.60 days), compared with the lower peak concentration (48.8 EUs) for HlyE IgA and later peak time (3.59 days).

To estimate the duration of elevated antibody responses to *Salmonella* Typhi or Paratyphi A infection, we compared the observed and modeled antibody decay curves to antibody threshold values identified in the cutpoint analysis (Appendix Table 8, Figure 2). By 90 days after symptom onset, 78% of IgA responses to HlyE and 69% of IgA responses to LPS had fallen below joint biomarker cutoffs (HlyE IgA cutoff 31.21 EUs; LPS IgA cutoff 18.05 EUs) and were classified as negative. The positivity threshold for LPS IgA did not change when combined with HlyE IgA. For HlyE IgA alone, because the individual biomarker threshold value (9.23 EUs) was lower than the joint biomarker threshold, antibody responses remained above the case identification cutoff for a longer time. At 90 days after symptom onset, 66% of cases still had HlyE IgA levels above the threshold. Raising the HlyE IgA cutoff value in the joint biomarker improved specificity by reducing false positives from longer-persisting antibody responses.

## Discussion

In this study, we evaluated a serologic assay for detecting enteric fever across 3 countries, Bangladesh, Nepal, and Pakistan, using well-characterized samples from blood culture–confirmed cases and febrile controls with laboratory-confirmed etiology. The combination of IgA responses to *Salmonella* Typhi LPS and HlyE met the proposed minimum World Health Organization target product profile threshold for sensitivity (>85%) and were within 1% of the specificity threshold (>90%) (*20*). Performance varied by geographic setting and age group. Our findings highlight the utility of context-specific validation when developing and deploying serologic tools for enteric fever diagnosis. 

Differences in biomarker performance across sites might reflect underlying epidemiologic and immunologic factors, including age distribution of cases, transmission intensity, and control group characteristics. Overall, accuracy was highest in Bangladesh and Pakistan; results were consistent with those from previous studies (*9*,*12*). Accuracy was lowest in Nepal, where enteric fever case-patients are older and force of infection is lower. The performance of the combined biomarkers differed from our previous study in Nepal in the same location (AUC 0.95 [95% CI 0.90–1.00]) (*9*). The Kathmandu Valley might have experienced changes in enteric fever transmission intensity since the previous study (*16*,*21*). 

The combined biomarkers performed particularly well in children <5 years of age from Dhaka, Bangladesh, the study region with the highest force of infection and clinical enteric fever incidence (*16*). In contrast, few samples from young children were collected in Nepal, and samples of controls from Pakistan in this age group were limited. In both Nepal and Pakistan, we observed a trend of improved accuracy with increasing age; the combined biomarkers performed best in the oldest age group. However, confidence intervals for age and site–specific AUCs overlapped and results were not statistically significant; it is possible that the variation occurred by random chance. 

We modeled longitudinal persistence of HlyE and LPS IgA to evaluate the duration of elevated antibody levels above cutpoints following acute infection. LPS IgA levels reach higher peaks and show faster decay rates, making it an effective marker for recent infections, especially in settings with frequent *Salmonella* Typhi exposure. Conversely, with slower decline and longer persistence, HlyE IgA is useful for seroepidemiologic studies, offering insights into past exposures with a longer-lasting immune signature (*15*,*22*). Combined with analytical approaches that infer time since infection, we have used HlyE IgA levels to characterize seroincidence across a range of transmission intensities (*15*,*23*).

We previously demonstrated that peak antibody responses to HlyE vary by age; that finding could partially explain better performance of LPS over HlyE, particularly at sites with higher forces of infection, such as in Bangladesh and Pakistan compared with Nepal (*15*). LPS is a T cell–independent antigen that can directly activate B cells without T cell help, generating a faster antibody response than protein antigens such as HlyE that require the adaptive immune system. Consequently, during a first infection, HlyE antibody responses develop more slowly. In lower-incidence sites like Nepal, patients may be more likely to seek care for their first infection. Thus, their HlyE IgA levels may be lower than in sites with a higher force of infection. Including HlyE IgA in the diagnostic tests provided marginal benefit over LPS IgA alone; most cases with elevated HlyE IgA also had a high concentration of LPS IgA. However, HlyE IgA was better at identifying *Salmonella* Paratyphi A cases, which might be advantageous in Asia, where Paratyphi A circulation is more common. Although we used *Salmonella* Typhi LPS for this assay, the LPS O12 antigen is identical among *Salmonella* groups A, B, and D. As such, cross-reactive antibodies bind both *Salmonella* Typhi and Paratyphi (*24*,*25*). In South Asia, prevalence of invasive nontyphoidal *Salmonella* (iNTS) infections is relatively low. In settings that have a large iNTS disease burden, particularly Africa, including HlyE IgA in a diagnostic panel may improve discriminatory power, given the potential for cross-reactivity with LPS because of their shared O-antigen. We have shown previously that HlyE IgA can better discriminate between those groups (*15*).

HlyE and LPS are not present in typhoid conjugate vaccine (TCV); thus, those biomarkers have diagnostic utility after TCV introduction. However, one limitation is they cannot distinguish between serovars. In our study sites, circulating *Salmonella* Typhi and Paratyphi A strains have different antimicrobial resistance patterns, which could influence clinical treatment decisions (*26*). TCV does not protect against paratyphoid infection; thus, it will be critical to understand serovar-specific disease burdens to inform introduction of the existing TCVs and forthcoming typhoid/paratyphoid combination vaccines, measure vaccine impact, and update treatment guidelines.

Although we enrolled a broad age range of case and control participants, we lacked equal representation across age categories at each site. Some age plus site–specific strata had small sample sizes, especially for controls, limiting our statistical power and precision. In addition, enteric fever case-patients were substantially younger than controls in Nepal and Pakistan, potentially reducing the apparent difference in biomarker levels between cases and controls. Because there is no fully sensitive reference standard for enteric fever, we selected blood culture–confirmed cases and febrile controls with confirmed alternative etiologies to reduce outcome misclassification. That design strengthens internal classification of cases and controls, but it may limit generalizability to unselected patients experiencing acute febrile illness. Further evaluation in prospectively enrolled febrile populations will be important to evaluate biomarker robustness and performance across age strata, serovars, and diverse study populations in intended-use settings. In selecting controls, we required laboratory confirmation of a non–enteric fever pathogen; however, blood culture has low sensitivity and was not performed for all controls (*27*). Consequently, some controls might have been co-infected with *Salmonella *Typhi and Paratyphi A, which could have reduced our specificity estimates. Further work evaluating the accuracy of these biomarkers in low-burden settings and in Africa where iNTS is common is necessary to assess the generalizability of our findings.

This study used a kinetic ELISA, requiring a specialized ELISA reader. The end goal of typhoid diagnostic development is an accurate, rapid test that can be administered and interpreted with basic infrastructure and training. Better tools are needed to identify enteric fever cases quickly, accurately, and inexpensively. Because the spread of antimicrobial-resistant *Salmonella* Typhi limits the therapeutic toolkit, an improved diagnostic would lead to more appropriate treatment and reduce time to effective care. A point-of-care test developed with the same antigens is usable at lower-level clinical facilities with minimal laboratory set-up and training (*28*); that dual-path, lateral-flow assay demonstrated very high accuracy (AUC 0.98) when tested on adult samples from Bangladesh and Nepal and performed nearly as well in follow-up studies on pediatric samples in Bangladesh (AUC 0.97) and across a broad age range in Pakistan (AUC 0.92) (*13*,*14*). 

In conclusion, enteric fever remains pervasive in many low-income settings and affects patients, healthcare systems, and communities. We demonstrated that the combination of IgA to *Salmonella* Typhi HlyE and LPS has excellent potential to distinguish enteric fever from other confirmed febrile illnesses. Integrating those biomarkers into point-of-care diagnostics or clinical surveillance platforms would enhance case detection and prevention efforts, especially in areas where the use of blood culture is limited.

This article was preprinted at https://medrxiv.org/cgi/content/short/2025.06.20.25329792v1.

Appendix Additional information about the use of *Salmonella* Typhi hemolysin E and lipopolysaccharide IgA to identify enteric fever cases, South Asia.
