# Supplementary material for: Use of Salmonella enterica Serovar Typhi Hemolysin E and Lipopolysaccharide IgA to Identify Enteric Fever Cases, South Asia
Source: Emerg Infect Dis. 2026 Aug;32(8):1221–30. doi: 10.3201/eid3208.250753 (PMC13426854; doi:10.3201/eid3208.250753)
Supplement: Appendix — Additional information about the use of Salmonella Typhi hemolysin E and lipopolysaccharide IgA to identify enteric fever cases, South Asia. [file 25-0753-Techapp-s1.pdf]

Article DOI: https://doi.org/10.3201/eid3208.250753

EID cannot ensure accessibility for supplementary materials supplied by authors. Readers who have difficulty accessing supplementary content should contact the authors for assistance.

Use of *Salmonella enterica* Serovar Typhi Hemolysin E and Lipopolysaccharide IgA to Identify Enteric Fever Cases, South Asia

Appendix

Detailed ELISA Methods

Plates were coated with recombinant hemolysin E (HlyE) (1 μg/mL) or *Salmonella* Typhi lipopolysaccharide (LPS) purified from strain Ty21a (2.5 μg/mL). Ty21a is an attenuated strain derived from *Salmonella* Typhi strain Ty2, which is safer to handle at scale and more amenable to commercial production. We have compared IgA reactivity to LPS isolated in-house from Ty21a and Ty2, and to commercially obtained *Salmonella* Typhi LPS from Sigma and found comparable seroreactivity (Appendix Figure 1). Plasma samples were added in duplicate at dilutions of 1:1000 (LPS) or 1:500 (HlyE). Goat anti-human IgA conjugated to horseradish peroxidase (Jackson ImmunoResearch) was used to detect bound antibodies and peroxidase activity was measured at 450nm by using o-phenylenediamine. The maximum slope of the reaction over a three-minute period was extracted; results were reported as ELISA units (EU), the average of blank-adjusted sample values (milli-units per minute) divided by the mean value of the triplicate blank-adjusted positive controls and multiplied by 100.

Detailed Statistical Methods

We used t-tests and chi-squared tests to compare case and control characteristics. We evaluated how well antibodies to HlyE IgA and LPS IgA were able to distinguish between cases and controls using ROC analysis. We assessed the performance of the biomarkers individually and in combination. To evaluate the combined performance of the biomarker pair, we used fitted values from a previously validated logistic regression model with anti-HlyE IgA and LPS IgA ELISA values as predictors (*9*). To compare the performance of a given classification rule between different population strata, AUCs were compared using an extension of DeLong's

method, using an unpaired two-sample t-test with unequal sample size and unequal variance (*18*).

Cutpoint Analysis

We conducted a cutpoint analysis for each biomarker individually and for the combination of both biomarkers. Individual cutpoints for each biomarker were identified by finding the threshold that maximized the Youden's J statistic (sensitivity + specificity – 1). Separately, we then calculated sample percentiles of the ELISA values for each biomarker and considered all 10,000 pairs of LPS and HlyE percentiles as cutpoints. For each pair of cutpoints, we classified samples as cases if the antibody concentration in the sample was greater than or equal to the corresponding cutpoint for *either* LPS IgA *or* HlyE IgA (or both). We then calculated the balanced accuracy ((sensitivity + specificity) / 2) for each pairwise combination of the percentiles and for the previously calculated individual biomarker Youden's cutoffs. We identified the combinations of anti-HlyE and LPS IgA ELISA values that yielded the maximum balanced accuracy (rounded to two significant digits). When multiple combinations yielded the same balanced accuracy, we selected the threshold value that maximized sensitivity.

Appendix Table 1. Diagnostic criteria and methods for alternative etiology febrile controls*

		Brand used		
Etiology	Diagnostic inclusion criteria	Bangladesh	Nepal	Pakistan
Dengue	Positive IgM serology OR NS1 antigen positive	SD. Biosensor Dengue IgM/IgG<br>SD. Biosensor NS1 Ag	**Biotrol** Laboratories Pvt. Ltd Dengue NS1 Ag+Ab Combo Kit	VIDAS DENGUE NS1, & IgM, (Biomerieux)
Malaria	RDT positive OR blood smear positive	Bioline Malaria Ag P.f/Pv, Abbott Diagnostics Korea Inc	Zephyr Biomedicals (Tulip Diagnostics), FalciVax Rapid Test for Malaria Pv/Pf. Ref: 503010025	Biolin Malaria Ag P.f/Pan (Abbott)<br><br>STANDARD Q Malaria P.f/Pan Ag (SD. Biosensor)
Scrub typhus	Positive IgM serology	NA‡	InBios Scrub Typhus Detect	NA
Other bacteremia	Positive blood culture† excluding likely contaminants (e.g., coagulase negative *Staph spp*s, *Micrococcus*, *Bacillus sp*p, etc)	BACTEC	BACTEC	BACTEC
COVID-19	Positive PCR or rapid antigen test	Novel Coronavirus (2019-nCoV) Nucleic Acid Diagnostic Kit (PCR-Fluorescence Probing) - Sansure kit<br>Thermofisher Tag-path covid-19 RT-qPCR<br>AccuPower® SARS-CoV-2 Real Time RT-PCR Kit - BIONEER	Perkin Elmer Inc, New Coronavirus Nucleic Acid Detection Kit. Ref: 2019-nCOV-PCR-AUS<br>Seegene Inc, Allplex 2019-nCoV Assay. Ref: RP10244Y<br>Maccura Biotechnology, SARS-CoV-2 Fluorescent PCR Kit. Ref: EGN7103109<br>Rapid Kit: SD. Biosensor Standard Q COVID-19 Ag Test Kit. Ref: 09COV30D	cobas 6800 system (Roche Diagnostics)

* NS1, nonstructural protein 1; PCR, polymerase chain reaction; RDT, rapid diagnostic test.
†Blood culture was performed from whole blood using automated culture systems (BACTEC, Becton Dickinson, Franklin Lakes, NJ, USA; BacTAlert 3D, BioMérieux, Marcy-l'Étoile, France).
‡Not applicable. Test not performed at this site

Appendix Table 2. Distribution of antibodies to HlyE and LPS IgA by site and age

Biomarker	Age category	Bangladesh Case (N = 411)	Bangladesh Control (N = 79)	Nepal Case (N = 155)	Nepal Control (N = 102)	Pakistan Case (N = 84)	Pakistan Control (N = 82)	All Case (N = 650)	All Control (N = 263)	p value*
		Median (Q1, Q3)								
HlyE IgA concentration										
	<5y	23.4 (9.3, 45.9)	1.6 (0.9, 3.4)	59.3 (12.4, 112.9)	0.8 (0.4, 1.1)	34.5 (19.0, 48.3)	1.2 (0.7, 6.2)	24.3 (10.1, 47.6)	1.5 (0.6, 3.4)	<0.001
	5–15y	32.0 (11.6, 73.2)	4.4 (0.8, 8.5)	8.3 (2.3, 38.2)	5.5 (1.8, 9.6)	39.8 (14.3, 105.0)	3.1 (2.0, 10.2)	29.6 (9.9, 73.9)	4.2 (1.1, 9.4)	<0.001
	16+y	274.7 (NA)	1.2 (NA)	24.2 (8.2, 50.2)	5.1 (3.3, 8.1)	31.2 (15.7, 138.9)	3.2 (1.7, 4.7)	25.0 (8.9, 59.5)	4.0 (2.4, 6.3)	<0.001
LPS IgA concentration	<5y	80.5 (33.6, 137.3)	0.8 (0.3, 3.7)	61.4 (38.3, 93.9)	1.4 (0.9, 1.8)	70.4 (24.8, 147.9)	3.7 (1.2, 20.3)	78.8 (31.1, 137.3)	1.2 (0.5, 3.7)	<0.001
	5–15y	83.7 (31.7, 167.5)	4.9 (2.0, 14.4)	106.1 (31.3, 232.1)	2.4 (1.7, 4.8)	216.5 (95.3, 482.0)	2.6 (0.9, 13.3)	97.1 (39.5, 208.4)	3.5 (1.3, 12.3)	<0.001
	16+y	237.8 (NA)	2.3 NA	108.9 (36.0, 223.9)	5.7 (3.5, 11.1)	183.5 (124.5, 337.0)	6.8 (4.6, 10.0)	133.8 (53.1, 258.1)	6.5 (3.8, 10.3)	<0.001

*p values estimated for the comparisons between all cases and controls

Appendix Table 3. Receiver Operating Characteristic analysis of IgA antibodies to HlyE and LPS IgA overall and stratified by age category and study site*

Age category (y)	N Case	N Control	HlyE IgA AUC (95% CI)	HlyE IgA Sensitivity (95% CI) at 90% Specificity	HlyE IgA Specificity (95% CI) at 90% Sensitivity	LPS IgA AUC (95% CI)	LPS IgA Sensitivity (95% CI) at 90% Specificity	LPS IgA Specificity (95% CI) at 90% Sensitivity	HlyE + LPS IgA AUC (95% CI)	HlyE + LPS IgA Sensitivity (95% CI) at 90% Specificity	HlyE + LPS IgA Specificity (95% CI) at 90% Sensitivity
All	650	263	0.87 (0.84, 0.89)	0.68 (0.60, 0.75)	0.58 (0.44, 0.69)	0.92 (0.90, 0.94)	0.85 (0.80, 0.89)	0.80 (0.68, 0.87)	0.93 (0.91, 0.95)	0.86 (0.82, 0.91)	0.86 (0.76, 0.91)
<5	208	66	0.94 (0.91, 0.97)	0.83 (0.64, 0.93)	0.83 (0.73, 0.92)	0.93 (0.90, 0.97)	0.86 (0.74, 0.96)	0.88 (0.77, 0.95)	0.96 (0.92, 0.99)	0.91 (0.83, 0.97)	0.91 (0.82, 0.98)
5–15	305	51	0.83 (0.77, 0.89)	0.58 (0.45, 0.76)	0.49 (0.33, 0.65)	0.88 (0.83, 0.94)	0.75 (0.33, 0.89)	0.73 (0.55, 0.86)	0.90 (0.84, 0.95)	0.81 (0.27, 0.89)	0.76 (0.61, 0.88)
16+	137	146	0.83 (0.77, 0.88)	0.66 (0.55, 0.74)	0.18 (0.09, 0.60)	0.93 (0.89, 0.96)	0.86 (0.80, 0.92)	0.82 (0.42, 0.94)	0.92 (0.88, 0.96)	0.86 (0.79, 0.92)	0.76 (0.24, 0.94)
Bangladesh											
Overall	411	79	0.92 (0.89, 0.95)	0.76 (0.60, 0.84)	0.75 (0.65, 0.84)	0.94 (0.91, 0.96)	0.82 (0.74, 0.91)	0.84 (0.71, 0.92)	0.96 (0.93, 0.98)	0.88 (0.82, 0.94)	0.89 (0.80, 0.95)
<5	180	50	0.94 (0.90, 0.97)	0.84 (0.72, 0.94)	0.84 (0.70, 0.94)	0.96 (0.93, 0.98)	0.92 (0.79, 0.97)	0.92 (0.80, 0.98)	0.97 (0.95, 0.99)	0.94 (0.86, 0.98)	0.94 (0.84, 1.00)
5–15	230	28	0.88 (0.83, 0.94)	0.64 (0.52, 0.83)	0.61 (0.39, 0.79)	0.89 (0.82, 0.96)	0.76 (0.13, 0.87)	0.68 (0.46, 0.86)	0.92 (0.87, 0.98)	0.83 (0.23, 0.92)	0.79 (0.61, 0.93)

Age category (y)	N		HlyE IgA			LPS IgA			HlyE + LPS IgA		
	Case	Control	AUC (95% CI)	Sensitivity (95% CI) at 90% Specificity	Specificity (95% CI) at 90% Sensitivity	AUC (95% CI)	Sensitivity (95% CI) at 90% Specificity	Specificity (95% CI) at 90% Sensitivity	AUC (95% CI)	Sensitivity (95% CI) at 90% Specificity	Specificity (95% CI) at 90% Sensitivity
16+	1	1	NA	NA	NA	NA	NA	NA	NA	NA	NA
Nepal											
Overall	155	102	0.74 (0.68, 0.80)	0.54 (0.41, 0.65)	0.12 (0.05, 0.24)	0.90 (0.86, 0.94)	0.85 (0.77, 0.91)	0.75 (0.23, 0.92)	0.89 (0.85, 0.94)	0.80 (0.72, 0.90)	0.65 (0.15, 0.89)
<5	4	7	NA	NA	NA	NA	NA	NA	NA	NA	NA
5–15	39	13	0.62 (0.45, 0.79)	0.31 (0.05, 0.59)	0.15 (0.00, 0.46)	0.83 (0.69, 0.97)	0.54 (0.15, 0.95)	0.38 (0.08, 0.92)	0.82 (0.68, 0.96)	0.64 (0.10, 0.90)	0.38 (0.00, 0.85)
16+	112	82	0.78 (0.71, 0.84)	0.59 (0.46, 0.70)	0.07 (0.00, 0.39)	0.92 (0.87, 0.96)	0.84 (0.76, 0.92)	0.78 (0.33, 0.93)	0.90 (0.86, 0.95)	0.83 (0.74, 0.91)	0.67 (0.09, 0.91)
Pakistan											
Overall	84	82	0.91 (0.87, 0.96)	0.82 (0.68, 0.92)	0.78 (0.39, 0.93)	0.94 (0.90, 0.98)	0.90 (0.77, 0.96)	0.90 (0.70, 0.98)	0.95 (0.92, 0.99)	0.93 (0.85, 0.99)	0.94 (0.80, 0.99)
<5	24	9	0.94 (0.86, 1.00)	0.75 (0.54, 1.00)	0.78 (0.44, 1.00)	0.77 (0.53, 1.00)	0.12 (0.00, 0.83)	0.56 (0.22, 0.89)	0.83 (0.63, 1.00)	0.29 (0.12, 0.96)	0.78 (0.33, 1.00)
5–15	36	10	0.82 (0.65, 0.99)	0.67 (0.08, 0.94)	0.60 (0.10, 0.90)	0.95 (0.88, 1.00)	0.94 (0.58, 1.00)	0.90 (0.60, 1.00)	0.93 (0.83, 1.00)	0.97 (0.39, 1.00)	0.90 (0.60, 1.00)
16+	24	63	0.92 (0.83, 1.00)	0.88 (0.67, 1.00)	0.86 (0.14, 0.98)	0.97 (0.92, 1.00)	0.96 (0.83, 1.00)	0.98 (0.40, 1.00)	0.96 (0.90, 1.00)	0.92 (0.79, 1.00)	0.98 (0.21, 1.00)

*AUC, Area Under the Curve; HlyE, Hemolysin E; LPS, Lipopolysaccharide, NA, Not applicable.

Appendix Table 4. Receiver Operating Characteristic analysis of IgA antibodies to HlyE and LPS IgA overall and stratified by serovar and study site*

Site	Serovar	N		HlyE IgA			LPS IgA			HlyE + LPS IgA		
		Case	Control	AUC (95% CI)	Sensitivity (95% CI) at 90% Specificity	Specificity (95% CI) at 90% Sensitivity	AUC (95% CI)	Sensitivity (95% CI) at 90% Specificity	Specificity (95% CI) at 90% Sensitivity	AUC (95% CI)	Sensitivity (95% CI) at 90% Specificity	Specificity (95% CI) at 90% Sensitivity
Overall	Typhi	568	263	0.86 (0.84, 0.89)	0.68 (0.59, 0.74)	0.56 (0.42, 0.67)	0.93 (0.91, 0.95)	0.87 (0.83, 0.90)	0.84 (0.77, 0.90)	0.94 (0.92, 0.95)	0.87 (0.83, 0.92)	0.87 (0.79, 0.92)
	Paratyphi A	82		0.90 (0.85, 0.94)	0.74 (0.61, 0.84)	0.73 (0.28, 0.84)	0.84 (0.78, 0.90)	0.72 (0.57, 0.82)	0.37 (0.16, 0.75)	0.90 (0.86, 0.95)	0.80 (0.71, 0.88)	0.70 (0.46, 0.89)
Bangladesh	Typhi	356	79	0.92 (0.89, 0.95)	0.76 (0.60, 0.83)	0.75 (0.63, 0.84)	0.95 (0.92, 0.97)	0.85 (0.78, 0.93)	0.86 (0.76, 0.94)	0.96 (0.94, 0.98)	0.90 (0.84, 0.96)	0.91 (0.82, 0.97)
	Paratyphi A	55		0.92 (0.87, 0.97)	0.76 (0.56, 0.91)	0.77 (0.42, 0.91)	0.87 (0.81, 0.93)	0.64 (0.44, 0.84)	0.54 (0.34, 0.81)	0.93 (0.88, 0.97)	0.78 (0.67, 0.91)	0.78 (0.59, 0.92)
Nepal	Typhi	129	102	0.71 (0.65, 0.78)	0.50 (0.35, 0.61)	0.11 (0.04, 0.20)	0.91 (0.87, 0.95)	0.86 (0.79, 0.93)	0.79 (0.23, 0.95)	0.89 (0.85, 0.94)	0.79 (0.70, 0.90)	0.65 (0.12, 0.88)
	Paratyphi A	26		0.87 (0.77, 0.97)	0.77 (0.58, 0.92)	0.61 (0.05, 0.94)	0.86 (0.75, 0.97)	0.77 (0.58, 0.92)	0.42 (0.04, 0.95)	0.89 (0.79, 0.99)	0.85 (0.69, 0.96)	0.44 (0.04, 0.98)
Pakistan	Typhi	83	82	0.91 (0.87, 0.96)	0.82 (0.67, 0.92)	0.78 (0.40, 0.93)	0.94 (0.90, 0.98)	0.90 (0.77, 0.96)	0.90 (0.72, 0.98)	0.95 (0.92, 0.99)	0.93 (0.86, 0.98)	0.94 (0.79, 0.99)
	Paratyphi A	1										

*AUC, Area Under the Curve; HlyE, Hemolysin E; LPS, Lipopolysaccharide; NA, Not applicable.

Appendix Table 5. Sensitivity analysis of the inclusion criteria for number of days of fever at clinical presentation

	Days of	Case	Control	HlyE IgA*		LPS IgA		HlyE + LPS IgA	
Site	Fever	(N)	(N)	AUC (95% CI)	p value	AUC (95% CI)	p value	AUC (95% CI)	p value
All	≤3	200	123	0.82 (0.78, 0.87)	ref	0.92 (0.89, 0.95)	ref	0.93 (0.90, 0.96)	ref
	4–5	223	69	0.89 (0.86, 0.93)	0.02	0.94 (0.92, 0.97)	0.22	0.96 (0.94, 0.98)	0.06
	6–14	227	71	0.89 (0.84, 0.93)	0.05	0.88 (0.84, 0.93)	0.18	0.91 (0.87, 0.95)	0.50
Bangladesh	≤3	120	47	0.87 (0.81, 0.93)	ref	0.94 (0.89, 0.98)	ref	0.94 (0.90, 0.99)	ref
	4–5	146	19	0.96 (0.93, 1.00)	0.01	0.96 (0.93, 0.99)	0.43	0.98 (0.97, 1.00)	0.09
	6–14	145	13	0.94 (0.89, 0.99)	0.07	0.90 (0.84, 0.96)	0.31	0.94 (0.90, 0.98)	0.93
Nepal	≤3	70	56	0.71 (0.62, 0.80)	ref	0.91 (0.85, 0.97)	ref	0.89 (0.83, 0.95)	ref
	4–5	48	19	0.74 (0.62, 0.85)	0.70	0.92 (0.84, 0.99)	0.91	0.92 (0.85, 0.99)	0.56
	6–14	37	27	0.81 (0.70, 0.92)	0.17	0.90 (0.81, 0.98)	0.81	0.89 (0.79, 0.98)	0.94
Pakistan	≤3	10	20	0.98 (0.95, 1.00)	ref	0.98 (0.94, 1.00)	ref	1.00 (1.00, 1.00)	ref
	4–5	29	31	0.90 (0.82, 0.99)	0.08	0.95 (0.89, 1.00)	0.51	0.96 (0.89, 1.00)	0.24
	6–14	45	31	0.90 (0.82, 0.97)	0.89	0.90 (0.83, 0.97)	0.29	0.92 (0.86, 0.99)	0.44

*HlyE, Hemolysin E; LPS, Lipopolysaccharide; AUC, Area Under the Curve.

Appendix Table 6. Sensitivity analysis of age inclusion criteria*

				HlyE IgA*		LPS IgA		HlyE + LPS IgA	
Site	Cohort	Case (N)	Control (N)	AUC	p value	AUC	p value	AUC	p value
All†	≤50y	650	263	0.87 (0.85, 0.89)	0.79	0.92 (0.90, 0.94)	0.84	0.93 (0.91, 0.95)	0.84
	No age restriction	652	301	0.86 (0.84, 0.89)		0.91 (0.89, 0.93)		0.93 (0.91, 0.95)	
Nepal	≤50y	155	102	0.74 (0.68, 0.80)	0.99	0.90 (0.86, 0.94)	0.93	0.89 (0.85, 0.94)	0.96
	No age restriction	157	121	0.74 (0.68, 0.80)		0.90 (0.86, 0.94)		0.89 (0.85, 0.93)	
Pakistan	≤50y	84	82	0.91 (0.87, 0.96)	0.87	0.94 (0.90, 0.98)	1.00	0.95 (0.92, 0.99)	1.00
	No age restriction	84	101	0.91 (0.86, 0.95)		0.94 (0.90, 0.98)		0.95 (0.92, 0.99)	

*HlyE, Hemolysin E; LPS, Lipopolysaccharide; AUC, Area Under the Curve.
†All sites includes Bangladesh. No participants in Bangladesh were >50 y.

Appendix Table 7. Modeled longitudinal kinetic parameter estimates

Parameter	Description	Units	HlyE IgA Median (Q1, Q3)	LPS IgA* Median, (Q1, Q3)
Decay Rate (α)	The rate at which antibody concentrations decline during the waning phase after the peak response.<br>-Smaller values indicate slower decay	Days	0.00031<br>(0.00015, 0.00062)	0.00035<br>(0.00011, 0.0010)
Shape Factor (r)	Describes the nonlinearity of antibody decay:<br><br>- When r>1, decay starts rapidly and slows over time, deviating from exponential decay<br>- Higher r values indicate faster early decay, transitioning to slower decay later	Dimensionless	2.07<br>(1.80, 2.40)	2.35<br>(2.01, 2.81)
Time to Peak (t1)	Represents the time taken to reach the maximum antibody concentration after symptom onset	Days	3.59<br>(2.10, 5.94)	2.60<br>(1.62, 4.14)
Baseline Antibody Concentration (y0)	Initial antibody concentration before infection	ELISA Units	4.34<br>(1.98, 10.49)	4.64<br>(2.44, 10.44)
Peak Antibody Concentration (y1)	Maximum antibody concentration achieved at peak	ELISA Units	48.80<br>(25.61, 94.13)	232.55<br>(125.95, 453.51)

*HlyE, Hemolysin E; LPS = Lipopolysaccharide.

Appendix Table 8. Performance of individual and joint biomarker cutpoints

Performance measure	Individual Biomarker		Joint Biomarker	
	HlyE IgA	LPS IgA	HlyE IgA	LPS IgA*
Cutoff (EU)	9.23	18.05	31.21	18.05
Sensitivity	0.76 (0.65, 0.85)	0.85 (0.78, 0.89)	0.88 (0.85, 0.92)	
Specificity	0.84 (0.71, 0.92)	0.90 (0.81, 0.96)	0.89 (0.84, 0.94)	
Balanced Accuracy	0.80	0.88	0.89	

*HlyE, Hemolysin E; LPS = Lipopolysaccharide.

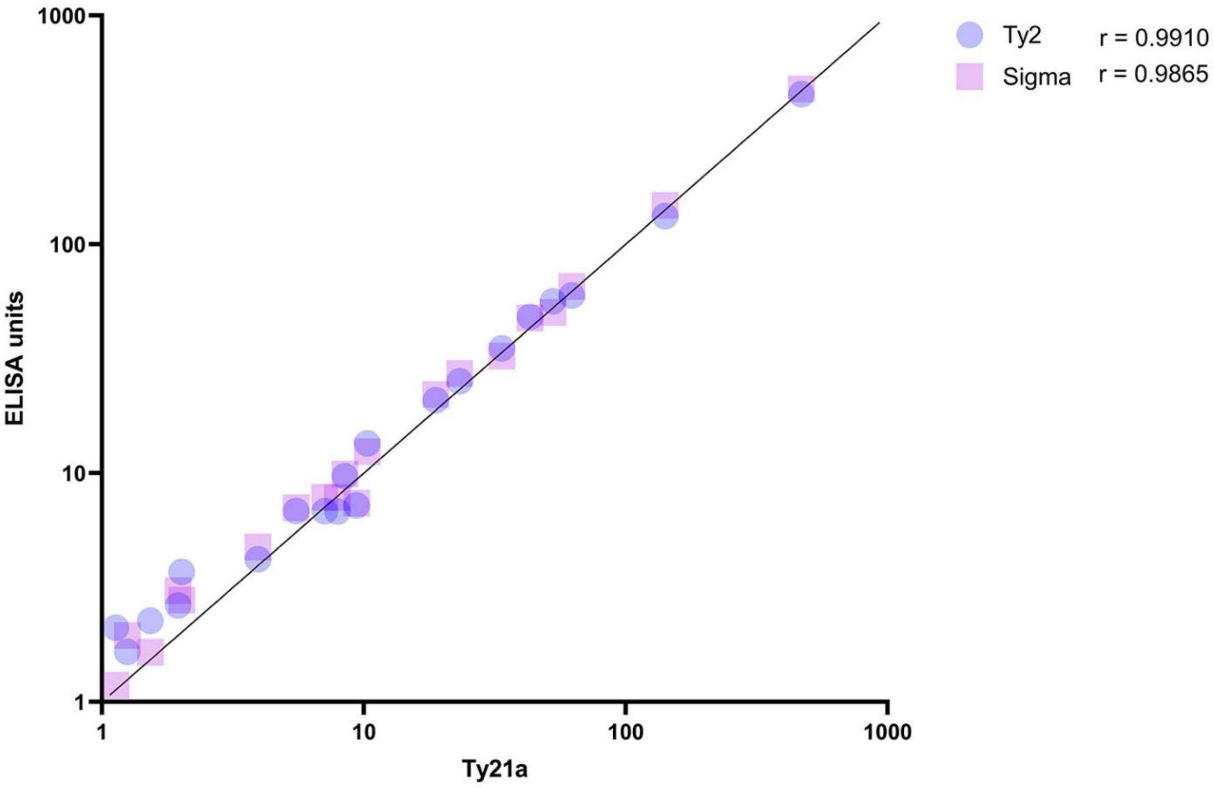

Appendix Figure 1. Comparison of IgA reactivity to *Salmonella* Typhi LPS isolated from Ty21a and Ty2 and commercially produced LPS (Sigma).

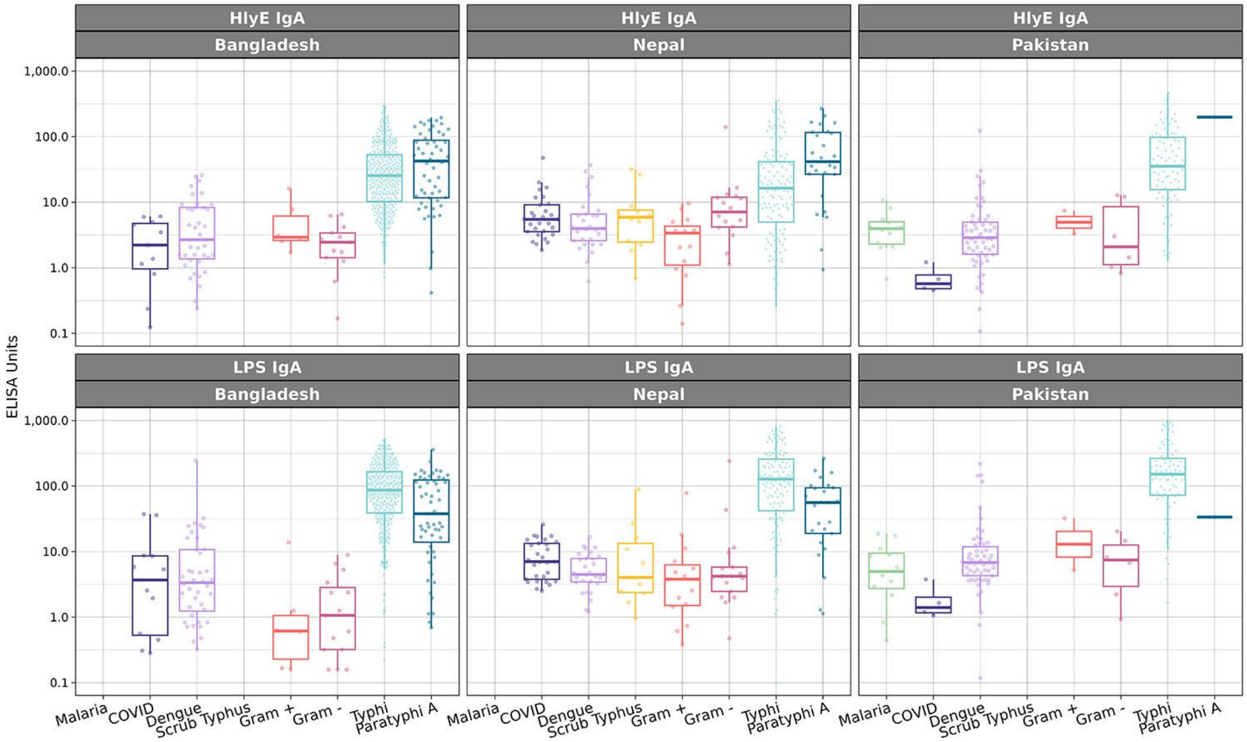

Appendix Figure 2. Distribution of anti-HlyE and LPS IgA antibodies among febrile cases by pathogen type and site. Boxplots show the distribution of plasma IgA responses against HlyE and LPS among *Salmonella* Typhi (Bangladesh n = 356; Nepal n = 129; Pakistan n = 83) and Paratyphi A (Bangladesh n = 55; Nepal n = 26; Pakistan n = 1) cases and alternative etiology febrile controls (dengue (Bangladesh n = 41; Nepal n = 29; Pakistan n = 58), COVID-19 (Bangladesh n = 12; Nepal n = 29; Pakistan n = 4), malaria (Pakistan n = 12), scrub typhus (*Orientia tsutsugamushi,* Nepal n = 12), gram-negative bacteria (Bangladesh n = 17; Nepal n = 17; Pakistan n = 6), gram-positive bacteria (Bangladesh n = 9; Nepal n = 15; Pakistan n = 2)).

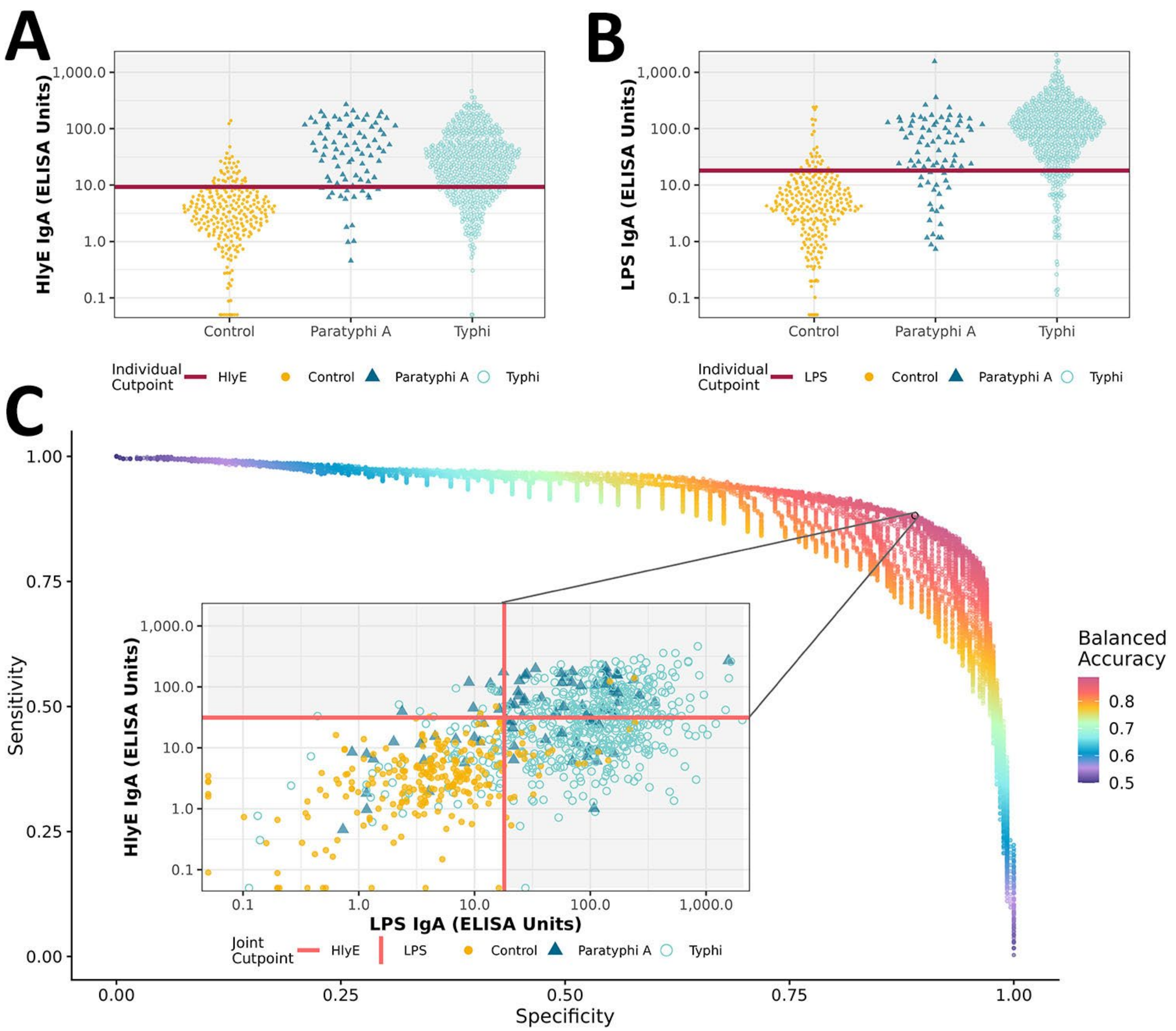

Appendix Figure 3. Cutpoint analysis. Plots A, B, and C show ELISA values for IgA antibodies to HlyE and LPS from Typhi and Paratyphi A cases and alternative etiology controls. Case identification thresholds are indicated by overlaid lines and values classified as cases are shown in the shaded gray area. Individual biomarker cutpoints selected by maximizing Youden's criteria are shown in A (HlyE) and B (LPS). For the joint biomarker cutpoint, sample generated percentiles of the ELISA values were calculated for each biomarker and all 10,000 pairs of LPS and HlyE percentiles were considered as joint cutpoints. For each pair of cutpoints, samples were classified as cases if the antibody concentration in the sample was greater than or equal to the corresponding cutpoint for *either* anti-LPS IgA *or* HlyE IgA (or both). The balanced accuracy (sensitivity + specificity) / 2) was calculated for each pairwise combination of the percentiles. In C, each cutpoint pair is shown as a circle on the larger plot with color indicating the balanced accuracy. The inset plot shows the optimal joint cutpoint and the classification rules can be visualized as rectangular classification boundaries.
